# Effectiveness and safety of adalimumab in patients with intestinal Behçet’s disease: a real-world prospective observational study in South Korea

**DOI:** 10.1186/s12876-023-03090-x

**Published:** 2023-12-19

**Authors:** Jongwook Yu, Sung Jae Shin, Yune-Jung Park, Hyung Wook Kim, Bo-In Lee, Byong Duk Ye, Geun-Tae Kim, Sung Kook Kim, Joo Sung Kim, Young-Ho Kim, Seonjeong Jeong, Jae Hee Cheon

**Affiliations:** 1 Department of Internal Medicine, Institute of Gastroenterology, Yonsei University College of Medicine, 50-1 Yonsei-ro, Seodaemun-gu, Seoul, 03722 Korea; 2 Department of Gastroenterology, Ajou University School of Medicine, Suwon, Korea; 3 Division of Rheumatology, Department of Internal Medicine, St. Vincent’s Hospital, The Catholic University of Korea, Suwon, South Korea; 4 Department of Internal Medicine, Pusan National University Yangsan Hospital, Yangsan, South Korea; 5 Division of Gastroenterology, Department of Internal Medicine, Seoul St. Mary’s Hospital, College of Medicine, The Catholic University of Korea, Seoul, Korea; 6 Department of Gastroenterology and Inflammatory Bowel Disease Center, Asan Medical Center, University of Ulsan College of Medicine, Seoul, South Korea; 7 Department of Internal Medicine, Kosin University College of Medicine, Busan, Korea; 8 Department of Internal Medicine, Kyungpook National University Hospital, Daegu, Korea; 9 Department of Internal Medicine and Liver Research Institute, Seoul National University College of Medicine, Seoul, Korea; 10 Departments of Medicine, Samsung Medical Center, Sungkyunkwan University School of Medicine, Seoul, Korea; 11 AbbVie Ltd, Seoul, Republic of Korea

**Keywords:** Behçet’s syndrome, Adalimumab, Inflammatory bowel Diseases, Tumor necrosis factor-alpha

## Abstract

**Background:**

Intestinal Behçet’s disease (BD) is characterized by typical gastrointestinal ulcers in patients with BD followed by complications such as bleeding, perforation and fistula. Biologic agents are currently under active investigation to delay the disease course. Various data regarding infliximab are available, but there is relatively lack of data regarding adalimumab.

**Methods:**

This was a multicenter, real-world prospective observational study to evaluate the effectiveness and safety of adalimumab in intestinal BD. The primary endpoint was disease activity at each follow up, including disease activity index for intestinal Behçet’s disease (DAIBD), serum C-reactive protein (CRP) level, and endoscopic findings. The secondary endpoint was the incidence of adverse drug reactions (ADRs).

**Results:**

A total of 58 patients were enrolled and 8 of them were excluded. Adverse events were reported in 72.0% of patients with 122 events. ADRs were reported in 24.0% with 28 events. For adverse events, arthralgia was most commonly reported (13.1%: 16/122) and only one experienced critical adverse event (0.82%, 1/122: death due to stroke). On multivariable regression analysis, a longer disease duration was significantly associated with decreased ADRs [Odds ratio 0.976 (0.953–0.999, 95% CI); *p =* 0.042]. Clinical response rates as assessed by DAIBD were 90.9% at Week 12 and 89.7% at Week 56, respectively. The mean serum CRP level at baseline was significantly decreased after 12 weeks (3.91 ± 4.93 to 1.26 ± 2.03 mg/dL; *p* = 0.0002).

**Conclusion:**

Adalimumab was found to be safe and effective in Korean patients with intestinal BD. A longer disease duration was significantly associated with decreased ADRs.

## Background

Behçet’s disease (BD) is a chronic immune-mediated systemic disease of unknown etiology and can involve multiple organs [1, 2]. Intestinal BD is characterized by typical gastrointestinal ulcers in patients with BD and is most prevalent in countries near the Silk Road [3, 4]. Endoscopically, oval-shaped, or round deep ulcers with discrete margin are usually found in the ileocecal area and complications such as bleeding, perforation and fistula frequently followed [2, 3]. Empirical medical treatments such as 5-aminosalicylic acids (5-ASA), immunomodulators or corticosteroids have been used to manage intestinal BD [5, 6]. However, for patients who are refractory about medical treatments, surgery can be inevitable but preventing and managing postoperative recurrence represents another important field of research in IBD [7–10]. A few biologic agents have been successfully used to delay the progression of intestinal BD and treat refractory cases [2–4, 11–15]. Among these agents, adalimumab is a recombinant, fully human IgG1 monoclonal antibody that specifically binds to tumor necrosis factor (TNF)-ɑ which showed durable long-term efficacy and safety in BD although there have been a few studies for intesitinal BD [16, 17]. It is one of the most commonly used biologics agents in inflammatory bowel disease such as Crohn’s disease and ulcerative colitis [18–20]. Furthermore, considering that TNF-ɑ is one of the main inflammatory mediators in intestinal BD, TNF-ɑ inhibitors might also be theoretically effective in intestinal BD. Recently, clinical studies investigating TNF-ɑ inhibitors for the management of intestinal BD have been increasing. In intestinal BD, various data regarding infliximab are available, but there is relatively lack of data regarding adalimumab even though adalimumab has been approved in South Korea in 2015 for the management of intestinal BD with post-marketing surveillance (PMS) study in progress [12, 13, 21–25]. In this study, we tried to prove the effectiveness and safety of adalimumab in Korean patients with intestinal BD for the first time using the PMS data.

## Methods

### Study design

This was a multicenter (ten tertiary medical centers in South Korea), prospective, real-world observational study to evaluate the effectiveness and safety of adalimumab in Korean patients with intestinal BD. Patients aged 19 years and older were enrolled from February 2016 (date of the first enrolled patient) to March 2020 (date of the last enrolled patient) in South Korea. The enrolled patients were regularly followed up until 56 weeks from the initial administration of adalimumab (Week 4, Week 8, Week 12, Week 28, and Week 56). All the adverse events and drug reactions were reported for the evaluation of safety. Effectiveness was evaluated with disease activity index based on questionnaire, serum inflammatory biomarkers and endoscopy during each point of follow up. Patients who were administered adalimumab for the diseases other than intestinal BD were excluded. In addition, patients who violated the standard administrative regimen of adalimumab were excluded. Patients who were lost during follow-up were also excluded.

### Study endpoints

The primary endpoints were disease activity during each follow up including disease activity index for intestinal BD (DAIBD), serum C-reactive protein (CRP) level, endoscopic findings, and change in extraintestinal manifestations. The secondary endpoint was the incidence of adverse drug reactions (ADRs).

### Administration of adalimumab

For the induction phase, an initial dose of 160 mg was administered subcutaneously over 1 or 2 days (Day 0), followed by 80 mg of adalimumab administered 2 weeks later (Day 15). For maintenance, 40 mg of adalimumab was administered every other week from Day 29.

### Assessment of safety

Safety of adalimumab was assessed for all patients who received at least one dose of adalimumab and were followed up. Information about safety profiles were collected to find adverse events and adverse drug reactions. Safety profiles such as adverse events, adverse drug reactions, serious adverse events, serious drug reactions and unexpected adverse events were defined according to the International Council for Harmonisation: clinical safety data management [26].

Medical histories, adverse events and adverse drug reactions were presented using the Medical Dictionary for Regulatory Activities (MedDRA 23.0), System Organ Class (SOC) and Preferred Term (PT). Adverse events and adverse drug reactions were further presented as events/100 patient-years of adalimumab exposure.

### Assessment of clinical outcomes

Clinical outcomes were assessed based on three objectives: DAIBD, serum CRP, endoscopic assessment, and change in extraintestinal manifestations.

#### DAIBD

The disease activity of intestinal BD was evaluated by DAIBD [27]. DAIBD was assessed at each point of visit (Baseline, Week 4, Week 8, Week 12, Week 28 and Week 56) and results were compared with the previous measurements. The effectiveness of adalimumab was assessed if DAIBD was decreased more than 20 points compared with DAIBD during the previous visit.

#### CRP

Serum CRP level using latex agglutination test was measured at each points of visit (Baseline, Week 4, Week 8, Week 12, Week 28 and Week 56) and an average was obtained.

#### Endoscopic assessment

Serial endoscopic assessments (Baseline, Week 28, Week 56) were compared with baseline endoscopic results (0: mucosa healing; 1: marked reduction of disease involvement; 2: reduction of disease involvement; 3: no change or aggravation of disease involvement) [28].

#### Extra-intestinal manifestations

At baseline, the percentages of having extra-intestinal symptoms in all patients were investigated and serial changes in the symptoms (improvement or no change or aggravation or unavailable) were followed up (Week 4, Week 8, Week 12, Week 28, and Week 56 or at the time of early termination). Aggravation or newly developed symptoms were classified as adverse events.

### Statistical analysis

Continuous variables are presented as means with standard deviation using paired t-test and Wilcoxon signed-rank test. Categorical variables are presented as absolute numbers and percentages. Univariable analysis was performed to identify factors associated with adverse events, adverse drug reactions and clinical response. For multiple logistic regression analysis using the stepwise method, the variables whose *p*-value ≤ 0.2 in simple logistic regression are considered as independent variables while variables with variance inflation factor (VIF) greater than 10 from the independent variable are excluded. Odds ratios and corresponding 95% confidence intervals were calculated. Results were considered statistically significant at *p*-values < 0.05.

### Ethical consideration

This study was performed in accordance with the ethical guidelines of the 1975 Declaration of Helsinki, and was approved by the Institutional Review Board of each participating hospital. All patients agreed to the conditions of the study and signed their informed consents.

## Results

### Baseline patient characteristics

A total of 58 patients were enrolled from Feb. 2016 to Mar. 2020. Nine of them were excluded from the analysis (5 due to inappropriate doses; 2 due to inclusion criteria violation; 1 due to follow up loss).

For the adalimumab regimen, the mean dosage per patients was 1508.8 ± 370.4 mg (from minimum of 240.00 mg to maximum of 2320.0 mg). The average number of administration per patient was 33.8 ± 9.2 times (from minimum of 2.0 to maximum of 54.0). During their final visits, 88.0% of the patients (44/50) maintained to use adalimumab, but 12.0% (6/50) stopped to receive adalimumab (3 due to researcher decision; 1 due to adverse events; 1 due to ineffectiveness; 1 due to unknown cause).

Among the patients, 44.0% (22/50) were men and the mean age was 47.5 ± 14.8 years. The mean disease duration was 57.1 ± 55.1 months (from minimum of 0 to maximum of 216.0). Among the 50 patients, 92% of patients (46/50) had experienced previous treatment for intestinal BD and 80% (40/50) of patients had experienced systemic steroids to control intestinal BD. For immunomodulatory medications, azathioprine was most frequently prescribed (27/50: 54%). In addition, biologic agents were previously used in 20% (10/50) of the patients and all cases used infliximab.

Extra-intestinal symptoms of BD presented in 72% of patients (36/50), with oral ulcer being the most commonly presented symptom (30/50: 60%). There was no case of active tuberculosis, but 2 patients had history of tuberculosis (2/50: 4%). Screening test for latent tuberculosis was performed in 76.0% (38/50) of patients, and 8 patients were treated for latent tuberculosis (6/8, 75.0%: isoniazid; 2/8, 25.0%: isoniazid and rifampin). Detailed patient baseline characteristics are presented in Table 1 (page 21).

**Table 1:** Patient baseline characteristics

	N = 50
Male, n (%)	22 (44.0)
Age (years)	47.5 ± 14.8^*^
Ever smoker, n (%)	14 (28.0)
Disease duration (months)	57.1 ± 55.1^*^
Extra-intestinal organ system involvement, n (%)^**^	36 (72.0)
History of tuberculosis, n (%)	2 (4.0)
Concomitant diseases, n (%) ^***^	36 (72.0)
Concomitant medications, n (%)	46 (92.0)
5-aminosalicylic acids or sulfasalazine	36 (72.0)
Systemic corticosteroids	29 (58.0)
Non-steroidal anti-inflammatory drugs	10 (20.0)
Immunosuppressants	22 (44.0)
Previous treatment for BD, n (%)	46 (92.0)
5-aminosalicylic acids and sulfasalazine, n (%)	42 (84.0)
Systemic corticosteroids, n (%)	40 (80%)
Immunomodulators, n (%)	
Azathioprine, n (%)	27 (54.0)
Methotrexate, n (%)	8 (16.0)
Previous biologics treatment, n (%)	10 (20.0)
Infliximab, n (%)	10 (20.0)

^*^mean ± standard deviation ^**^Oral ulcer (n = 30, 60.0%), genital ulcer (n = 6, 12.0%), ocular lesion (n = 8, 16.0%), skin lesion (n = 13, 26.0%), arthralgia (n = 11, 22.0%), vascular involvement (n = 0, 0%), central nervous system involvement (n = 1, 2.0%), others, (n = 2, 4.0%) ^***^Hypertension, dyslipidemia, diabetes mellitus, gout, hyperthyroidism, polycystic ovarian diseases, atrial flutter, gastric ulcer, gastroesophageal reflux diseases, osteopenia, rheumatoid arthritis, osteoarthritis, ulcerative colitis, Crohn’s disease, anemia, thrombocytopenia

### Safety of adalimumab

A total of 122 adverse events were reported in 72.0% (36/50) of the patients, and adverse drug reactions were reported in 24.0% (12/50) with 28 events (Table 2, page 24). A total of 37 serious adverse events were reported in 26.0% (13/50) and serious drug reactions were reported in 8.0% (4/50) with 11 events. Gastrointestinal system was most commonly affected (7 patients for 20 serious adverse events) and infection was the secondly reported as serious adverse events (6 patients for 7 events). Infection was the most commonly reported serious drug reactions (3 patients for 3 events). Unexpected adverse events were reported 54.0% (27/50) with 55 events and unexpected adverse drug reactions were 14.0% (7/50) with 9 events (Table 2, page 24). Event rates as assessed by events per 100 patient-years were 201.7 for adverse events, 46.3 for adverse drug reactions, 61.2 for serious adverse events, 18.2 for adverse drug reactions, 90.9 for unexpected adverse events and 14.9 for unexpected adverse drug reactions, respectively.

**Table 2:** Adverse events and adverse drug reactions

Safety events	Number of patients, n (%)	Number of events, n (%)	Event rate^*^
Any AEs	36 (72.0)	122 (100)	201.7
Serious AEs	13 (26.0)	37 (30.3)	61.2
Unexpected AEs	27 (54.0)	55 (45.1)	90.9
Organ systems commonly affected by AEs^**^			
Gastrointestinal disorders	21 (42.0)	41 (33.6)	67.8
Skin and subcutaneous disorders	15 (30.0)	17 (13.9)	28.1
Musculoskeletal disorders	14 (28.0)	16 (13.1)	26.5
Infectious disorders	12 (24.0)	14 (11.5)	23.1
Any ADRs	12 (24.0)	28 (23.0)	46.3
Serious ADRs	4 (8.0)	11 (9.0)	18.2
Unexpected ADRs	7 (14.0)	9 (7.4)	14.9
ADRs of interest			
Infections^***^	4 (8.0)	4 (3.3)	6.6
Tuberculosis^****^	1 (2.0)	1 (0.8)	1.7

MedDRA 23.0 System Organ Class (SOC), Preferred Term (PT) AEs: adverse events; ADRs: adverse drug reactions; ^*^Event rate: events per 100 patient-years ^**^More than 20 events per 100 patient-years ^***^Infections by ADRs: acute pyelonephritis, disseminated tuberculosis, fungal infection, acute pharyngitis ^****^Adalimumab was discontinued at the time of diagnosis of disseminated tuberculosis and the patient completely recovered

For adverse events, musculoskeletal disorders (arthralgia) were most frequently reported (28.0%: 14/50) followed by oral ulcers (22.0%: 11/50) and skin lesions (20.0%: 10/50). Among 122 events, 6 events (4.9%) were classified as severe, 11 events (9.0%) were moderate, and the rest of them were classified as mild (86.1%). For the management of adverse events, adalimumab was permanently discontinued in 7.4% (9/122) and temporarily discontinued in 1.6% (2/122). Although 79.5% (97/122) of patients were completely recovered, 13 out of 122 (10.7%) did not recover. One patient experienced critical adverse events (0.8%, 1/122: death due to brain hemorrhage).

Univariable analysis (Chi-square test or Fisher’s exact test) and multivariable logistic regression model were carried out to determine factors associated with adverse events, and the results did not show statistically significant factors (Table 3, page 26). On the other hand, for adverse drug reactions, a longer disease duration and previous treatment for BD were associated with decreased risk based on the univariable analysis [Table 4, page 28; OR 0.973 (0.949–0.997, 95% CI) *p* = 0.026 for disease duration; OR 0.081 (0.008–0.874, 95% CI) *p* = 0.038 for previous treatment history]. Multivariable logistic regression was conducted with factors of *p* ≤ 0.2 in univariable analysis and a longer disease duration was significantly associated with decreased adverse drug reactions [Table 4, page 28; OR 0.976 (0.953–0.999, 95% CI) *p =* 0.042]. In this study, patients aged more than 65 were 14.0% (7/50) with adverse events in 71.4% (5/7) and adverse drug reactions in 14.3% (1/7). Skin lesions were most frequently reported (57.1%: 4/7).

**Table 3:** Logistic regression analysis for adverse events

	Univariable analysis		Multivariable analysis^*^		
Factors	Odds ratio (95% CI)	*p*-value	Odds ratio (95% CI)		*p*-value
Male	0.477 (0.136–1.670)	0.247			
Age (ref. ≥ 70 years)		0.621			
19 ~ 29 years	1.000 (0.045–22.175)				
30 ~ 39 years	6.000 (0.183-196.271)				
40 ~ 49 years	2.250 (0.111–45.723)				
50 ~ 59 years	4.500 (0.190-106.823)				
60 ~ 69 years	3.500 (0.145–84.694)				
Smoking history	1.527 (0.350–6.672)	0.679			
Oral ulcer	0.778 (0.217–2.793)	0.700			
Genital ulcer	0.750 (0.121–4.640)	0.757			
Ocular lesion	1.200 (0.212–6.801)	0.837			
Skin lesion	2.640 (0.504–13.835)	0.251			
Arthralgia	5.000 (0.576–43.388)	0.144	5.000 (0.576–43.388)	0.144	
Previous treatment history	0.846 (0.081–8.894)	0.889			
Previous medical history	1.833 (0.429–7.836)	0.413			
Concomitant diseases	2.625 (0.702–9.809)	0.151			
Concomitant medications	2.832 (0.358–22.386)	0.324			
Disease duration (months)	1.0005 (0.989–1.012)	0.937			
Total amount of adalimumab (mg)	1.0001 (0.998–1.002)	0.916			
Frequency of adalimumab administration (times)	1.004 (0.939–1.074)	0.900			

^*^Multivariable logistic regression analysis using stepwise method

**Table 4:** Logistic regression analysis for adverse drug reactions

	Univariable analysis		Multivariable analysis*	
Factors	Odds ratio (95% CI)	*p*-value	Odds ratio (95% CI)	*p*-value
Male	0.556 (0.143–2.162)	0.397		
Age (ref. ≥ 70 years)		0.178		
19 ~ 29 years	0.143 (0.004–4.612)			
30 ~ 39 years	1.333 (0.057–31.121)			
40 ~ 49 years	0.083 (0.003–2.603)			
50 ~ 59 years	0.571 (0.028–11.849)			
60 ~ 69 years	0.125 (0.004–3.996)			
Smoking history	1.336 (0.334–5.343)	0.881		
Oral ulcer	1.454 (0.373–5.679)	0.590		
Genital ulcer	1.701 (0.271–10.686)	0.571		
Ocular lesion	2.200 (0.440-11.006)	0.337		
Skin lesion	4.429 (1.095–17.915)	0.037	3.205 (0.576–17.827)	0.183
Arthralgia	3.810 (0.901–16.100)	0.069	5.581 (0.894–34.855)	0.066
Previous treatment for BD	0.081 (0.008–0.874)	0.038	0.062 (0.002–1.682)	0.099
Previous medical history	0.722 (0.165–3.157)	0.666		
Concomitant diseases	0.714 (0.176–2.898)	0.638		
Concomitant medications	0.278 (0.035–2.227)	0.228		
Disease duration (a month)	0.973 (0.949–0.997)	0.026	0.976 (0.953–0.999)	0.042
Total amount of adalimumab (mg)	0.999 (0.997–1.001)	0.251		
Frequency of adalimumab administration (times)	0.963 (0.900–1.030)	0.272		

^*^Multivariable logistic regression analysis using stepwise method

### Effectiveness of adalimumab

#### DAIBD

DAIBD was assessed during every visit and clinical response was defined if the score was decreased more than 20 points than the previous visit. [27] Clinical response rates were 81.8% (27/33) at Week 4, 87.5% (35/40) at Week 8, 90.9% (30/33) at Week 12, 90.9% (27/30) at Week 28, and 89.7% (26/29) at Week 56. The mean DAIBD was 109.3 ± 32.5 before administration of adalimumab, and after 12 weeks of administration, the mean DAIBD was decreased to 47.6 ± 43.7 (*p*<0.0001, Table 5). However, in cases of early termination (N = 2), DAIBD was not improved and the mean DAIBD was 110.0 ± 21.2 (95.0-125.0). For the patients who already received infliximab (N = 10), clinical response rates were 75.0% (6/8) at Week4, 90.0% (9/10) at Week 8, 66.7% (6/9) at Week 12, 88.9% (8/9) at Week 28, and 88.9% (8/9) at Week 56. The mean DAIBD was 104.0 ± 40.9 at baseline, and after adalimumab administration, it decreased to 49.3 ± 62.5 (*p =* 0.039) at Week 12, and 20.0 ± 23.5 (*p* = 0.008) at Week 56, respectively.

#### Serum CRP level

The mean serum CRP level (normal range of less than 0.9 mg/dL) was 3.91 ± 4.93 mg/dL and decreased to 1.26 ± 2.03 mg/dL after 12 weeks of administration (*p* = 0.0002, Table 5). At Week 56, the mean CRP level was 1.20 ± 2.46 mg/dL (*p* = 0.0006, Table 5). On the other hand, for cases of early termination (N = 4), the mean CRP level was increased to 4.25 ± 2.94 mg/dL. For the patients who already received infliximab (N = 10), the mean CRP level was 3.48 ± 2.58 (N = 8) and decreased to 1.50 ± 2.44 (*p* = 0.195) at Week 12, to 0.63 ± 0.60 (*p* = 0.023) at Week 56.

**Table 5:** Serial changes in DAIBD, CRP level and clinical response

Time point	DAIBD	Clinical response, n (%)^*^	CRP
Baseline	109.3 ± 32.5 (N = 48)		3.91 ± 4.93 (N = 44)
Week 4	50.3 ± 39.1 (N = 33)^**^	27 (81.8)	2.20 ± 4.02 (N = 38)^**^
Week 8	39.3 ± 36.1 (N = 40)^**^	35 (87.5)	1.41 ± 3.33 (N = 39)^**^
Week 12	47.6 ± 43.7 (N = 33)^**^	30 (90.9)	1.26 ± 2.03 (N = 43)^**^
Week 28	35.8 ± 37.1 (N = 30)^**^	27 (90.0)	1.08 ± 2.18 (N = 40)^**^
Week 56	33.5 ± 29.3 (N = 29)^**^	26 (89.7)	1.20 ± 2.46 (N = 40)^**^
Early termination	110.0 ± 21.2 (N = 2)	0 (0.0)	4.25 ± 2.94 (N = 4)
**Serial changes in patients who were previously treated with infliximab**			
Baseline	104.0 ± 40.9 (N = 10)		3.48 ± 2.58 (N = 8)
Week 4	40.6 ± 29.6 (N = 8)^**^	6 (75)	1.43 ± 1.97 (N = 10)
Week 8	32.0 ± 30.9 (N = 10)^**^	9 (90)	0.36 ± 0.43 (N = 8)^**^
Week 12	49.3 ± 62.5 (N = 9)^**^	6 (66.7)	1.50 ± 2.44 (N = 10)
Week 28	23.3 ± 32.4 (N = 9)^**^	8 (88.9)	0.51 ± 0.63 (N = 9)^**^
Week 56	20.0 ± 23.5 (N = 9)^**^	8 (88.9)	0.63 ± 0.60 (N = 10)^**^

^*^Clinical response is defined if the DAIBD score decreased more than 20 points than in the previous visit. ^**^A statistically significant decrease (*p* < 0.05) compared to baseline value

#### Endoscopic findings

At Week 28, 80.0% (4/5) achieved complete mucosa healing, and 20.0% (1/5) achieved marked reduction (more than 50% in ulcers) in disease extent assessed by endoscopy. At Week 56, 28.6% (2/7) achieved complete mucosa healing, reduction and no change or worse, respectively. 14.3% (1/7) achieved marked reduction in disease extent assessed by endoscopy.

#### Assessment of extra-intestinal symptoms

**Fig. 1:**
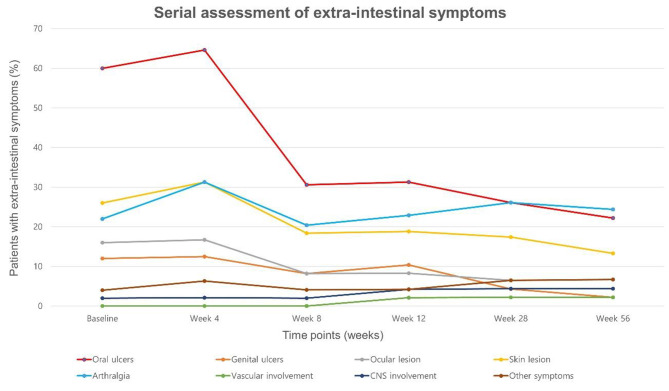
Serial assessment of extra-intestinal symptoms

The proportions of having extra-intestinal symptoms, especially oral ulcers, genital ulcers, ocular and skin lesions steadily decreased as time passed (Fig. 1). For arthralgia, the proportions did not decrease at each visit and new onset of arthralgia was frequently reported. For vascular and CNS involvement, one patient newly developed symptoms by Week 12.

In terms of clinical response, there were no significantly associated factors when assessed using univariable analysis and univariable logistic regression. Multivariable logistic regression analysis was carried out with factors of *p* ≤ 0.2 in the univariable analysis and no significantly associated factor was found (Table 6, page 30).

**Table 6:** Logistic regression analysis for clinical response

	Univariable analysis		Multivariable analysis^*^	
Factors	Odds ratio (95% CI)	*p*-value	Odds ratio (95% CI)	*p*-value
Male (ref. female)	0.408 (0.086–1.938)	0.260		
Age (ref. ≥ 70 years)		0.892		
19 ~ 29 years	0.520 (0.009–29.191)			
30 ~ 39 years	0.866 (0.013–56.376)			
40 ~ 49 years	1.666 (0.027-101.905)			
50 ~ 59 years	0.486 (0.009–24.964)			
60 ~ 69 years	1.133 (0.018–71.501)			
Smoking history	0.259 (0.052–1.285)	0.148		
Oral ulcer	0.173 (0.020–1.532)	0.115		
Genital ulcer	0.946 (0.095–9.378)	0.962		
Ocular lesion	1.400 (0.148–13.236)	0.769		
Skin lesion	2.800 (0.310-25.255)	0.359		
Arthralgia	0.818 (0.140–4.764)	0.823		
Previous treatment for BD	6.668 (0.785–56.652)	0.082	6.668 (0.785–56.653)	0.082
Previous medical history	3.498 (0.391–31.289)	0.263		
Concomitant medical diseases	3.200 (0.674–15.186)	0.143		
Concomitant medications	6.668 (0.785–56.652)	0.082		
Disease duration (months)	1.005 (0.989–1.021)	0.524		
Total amount of adalimumab (mg)	1.001 (1.000-1.003)	0.130		
Frequency of adalimumab administration (times)	1.060 (0.984–1.142)	0.127		

^*^Multivariable logistic regression analysis using stepwise method

## Discussion

Intestinal BD is a rare disease, but severe complications commonly follow if the disease is not well controlled. At some point in the course of the disease, surgery is required if medical management fails. Utmost effort to avoid surgery is required, and biologic agents have been used at earlier stages than before to delay disease progression in the management of inflammatory bowel diseases. Such preemptive strategies are also applicable in the management of intestinal BD. Infliximab has already shown its effectiveness and safety in intestinal BD, while adalimumab showed promising results even though well designed studies are limited to a few countries studying the small number of patients [3, 4, 22, 23, 25, 29–33]. Recently, Zhang et al. reported that adalimumab was effective and safe in a systematic review and meta-analysis although clinical response rate was lower than our study [Partial response rate (a decrease of more than 20 points in DAIBD score from the baseline or a significant improvement of symptoms assessed by investigators: 45% (95%CI 28–73%) at Month 6, 60% (95%CI 42–86%) at Month 12, 40% (95%CI 23–68%) at Month 24] [16]. In Japan, prospective studies showed that adalimumab was effective and safe in intestinal BD both in the short- and long-term period [21, 22, 24]. Most recently, the prospective, all-case, post-marketing study by Suzuki et al. reported that adalimumab was safe and effective in patients with intestinal BD (462 patients for a mean of 515.3 days in the safety population and 383 patients for a mean of 579.5 days in the effectiveness population) [21]. For the safety issue, adverse drug reactions and serious adverse drug reactions were reported in 26.0% and 11.0% of patients, respectively. The authors evaluated the effectiveness using global improvement rating and endoscopic assessment. Adalimumab was regarded effective overall in 324 patients (84.6%), and the response rates were higher than in previous studies [12, 22–25]. Although this prospective study with a large volume of patients for long term showed meaningful results, tools to assess effectiveness based on variables such as global gastrointestinal symptoms were subjective and serum CRP level at baseline was relatively low and even in nearly normal range (1.96 mg/dL) [21].

On the other hand, in this study we assessed the effectiveness of adalimumab with DAIBD, a more objective index that also includes extraintestinal symptoms [27]. Clinical response rates assessed by DAIBD were remarkable both in short- and long- term periods (90.9% at Week 12, 89.7% at Week 56, respectively). Based on similar clinical response rates in short- and long-term periods in our study, early response to adalimumab might be an important predictive marker for eventual drug response. In actual practice, insurance policy in South Korea covers adalimumab in patients with intestinal BD only when DAIBD at Week 12 decreased more than 20 points compared to baseline DAIBD. Additionally, serum CRP level was higher (3.91 ± 4.93 mg/dL) than in the previous study [21]. Intestinal BD is an intestinal manifestation of systemic BD, and careful evaluation and management of extra-intestinal disease symptoms such as skin lesions, uveitis, oral and genital ulcers are required when managing intestinal BD [34]. We also investigated extra-intestinal symptoms during every visit and followed the status of extra-intestinal symptom changes to integrate extra-intestinal symptoms into the systematic effect of adalimumab in intestinal BD. Though most extra-intestinal symptoms improved with adalimumab, the proportion of having arthralgia was not significantly decreased. In patients with IBD who were treated with TNF-ɑ inhibitors, paradoxical arthritis as a form of synovitis has been described after administration of TNF-ɑ inhibitors. In case of intolerable arthralgia, changing medications other than TNF-ɑ inhibitors should be considered [35–37].

In the sub-analysis for the patients who had previous infliximab treatment (N = 10), adalimumab showed statistically significant improvement in terms of clinical response and serum CRP level in both short-term and long-term follow-ups. These results suggest that adalimumab is effective in patients who are intolerable or refractory to infliximab. The measurement of serum drug level and antibody titer could provide detailed information about why infliximab failed in previous cases.

Most importantly, in this PMS study, we investigated detailed information about adverse drug reactions from adalimumab at every follow-up. Adverse drug reactions and serious adverse drug reactions were reported in 24.0% and 8.0% of patients, respectively, which were slightly lower than the previous study [21]. For safety evaluation, adalimumab proved to be very safe, although there was one case of mortality by subarachnoid hemorrhage, which was not considered to be associated with the administration of adalimumab. In multiple logistic regression analysis, no statistically meaningful factors were present for the drug adverse events, but for drug adverse reactions, a longer disease duration was a statistically significant factor for having lower drug adverse reactions. Previous exposure to immunomodulators and steroids in patients with longer disease duration might have reduced the immunogenicity to adalimumab.

To our knowledge, this is the first real-world, observational study that proved the safety and effectiveness of adalimumab in patients with intestinal BD in South Korea where the prevalence of intestinal BD is relatively higher than other countries. There are some limitations in this study. Firstly, the number of enrolled patients is not large enough even considering the rarity of this disease. However, the number can be comparable to that obtained by previous studies in other countries. Secondly, the drug level and antibody test for adalimumab could not be performed. Thirdly, the number of patients who received colonoscopy was also very small. Lastly, corticosteroids were used in 29 patients (58.0%) but detailed information about steroid-tapering was not available.

## Conclusion

Adalimumab was safe and effective in Korean patients with intestinal BD. Longer disease duration was significantly associated with the reduction in incidence of adverse drug reactions. Adalimumab remained effective for patients with intestinal BD who had already received infliximab.

## Data Availability

All data generated or analysed during this study are included in this published article.

## Abbreviations

BD: Behçet’s disease
ADRs: adverse drug reactions
DAIBD: disease activity index for intestinal Behçet’s disease
CRP: C-reactive protein
5-ASA: 5-aminosalicylic acids
TNF: tumor necrosis factor
